# P-2218. Evaluating the Timing of Bronchoscopy and Plasma Microbial Cell-Free DNA (mcfDNA) Sequencing on Diagnostic Yield: A Secondary Analysis of the Pneumonia in the ImmunoCompromised – use of the Karius Test for detection of Undiagnosed Pathogens (PICKUP) Study

**DOI:** 10.1093/ofid/ofae631.2372

**Published:** 2025-01-29

**Authors:** Ahmad Mourad, Daniel Lupu, Morgan M Richey, Vance G Fowler, Bradley A Perkins, Thomas L Holland, Stephen P Bergin

**Affiliations:** Duke University School of Medicine, Durham, North Carolina; Karius Inc, Redwood City, California; Karius, Inc, Redwood, California; Duke University Medical Center, Durham, NC; Karius Inc, Redwood City, California; Duke University Medical Center, Durham, NC; Duke University Health System, Durham, North Carolina

## Abstract

**Background:**

Delayed bronchoscopy has been associated with reduced diagnostic yield in immunocompromised patients with pneumonia. We sought to evaluate whether timing of bronchoscopy was associated with diagnostic yield, and if timing of plasma mcfDNA sequencing impacted additive diagnostic value, among patients enrolled in the PICKUP study (NCT04047719).

**Figure:**
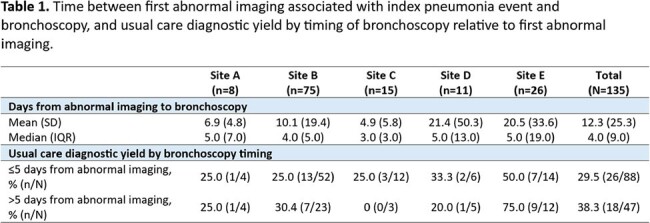

**Methods:**

The PICKUP study prospectively enrolled immunocompromised patients undergoing bronchoscopy to identify pneumonia etiology across ten US sites to establish whether plasma mcfDNA increased diagnostic yield compared to usual care. In this secondary analysis, pneumonia onset was defined as the first abnormal chest imaging associated with the index pneumonia event. Diagnostic yield and additive diagnostic value, defined as the patients with a probable cause of pneumonia exclusively identified by plasma mcfDNA sequencing, for those undergoing early (≤5 days from pneumonia onset) versus late ( >5 days) bronchoscopy was evaluated.

**Results:**

Of the 222 eligible patients enrolled between 01/2020 - 02/2022, we included patients from the first five sites in this preliminary analysis (n=135). Among the five included sites in this analysis, median time from pneumonia onset to bronchoscopy was 4.0 (IQR 9.0) days (Table 1). There was variability in diagnostic yield of usual care between the sites (range 20.0% - 61.5%). Usual care diagnostic yield was 26/88 (30%) for patients undergoing early and 18/47 (38%) for late bronchoscopy, p=0.30 (Table 1). Plasma mcfDNA was performed on the same day as bronchoscopy for most patients, median 0 (IQR 1.0) days. Plasma mcfDNA diagnostic yield was 19/88 (22%) for patients undergoing early versus 10/47 (21%) for late bronchoscopy, p=0.97. Additive diagnostic value of plasma mcfDNA was 9/88 (10%) for patients undergoing early versus 4/47 (9%) for late bronchoscopy, p=0.75.

**Conclusion:**

In this preliminary analysis, bronchoscopy timing was not associated with significant differences in diagnostic yield of usual care or additive diagnostic value of plasma mcfDNA sequencing. Ongoing analyses will evaluate whether variation in diagnostic yield between sites is associated with differences in patient characteristics or extent of diagnostic testing obtained.

**Disclosures:**

Daniel Lupu, MD, PHD, Karius, Inc: Employee|Karius, Inc: Stocks/Bonds (Private Company) Morgan M. Richey, PhD, Karius, Inc: Employee|Karius, Inc: Stocks/Bonds (Private Company) Vance G. Fowler, MD, MHS, Affinergy: Advisor/Consultant|ArcBio: Stocks/Bonds (Private Company)|Armata: Advisor/Consultant|Astra Zeneca: Advisor/Consultant|Astra Zeneca: Grant/Research Support|Basilea: Advisor/Consultant|Basilea: Grant/Research Support|ContraFect: Advisor/Consultant|ContraFect: Grant/Research Support|Debiopharm: Advisor/Consultant|Destiny: Advisor/Consultant|EDE: Grant/Research Support|Genentech: Advisor/Consultant|Genentech: Grant/Research Support|GSK: Advisor/Consultant|Janssen: Advisor/Consultant|Karius: Grant/Research Support|MedImmune: Grant/Research Support|Merck: Grant/Research Support|sepsis diagnostics: Patent pending|UptoDate: Royalties|Valanbuio: Stocks/Bonds (Private Company)|Valanbuio: Stocks/Bonds (Private Company) Bradley A. Perkins, MD, Karius, Inc: Employee|Karius, Inc: Stocks/Bonds (Private Company) Thomas L. Holland, MD, Karius, Inc: Advisor/Consultant

